# Digital Health for Migrants, Ethnic and Cultural Minorities and the Role of Participatory Development: A Scoping Review

**DOI:** 10.3390/ijerph20206962

**Published:** 2023-10-23

**Authors:** Irina Radu, Mandy Scheermesser, Martina Rebekka Spiess, Christina Schulze, Daniela Händler-Schuster, Jessica Pehlke-Milde

**Affiliations:** 1 Institute of Midwifery and Reproductive Health, School of Health Sciences, ZHAW Zurich University of Applied Sciences, Katharina-Sulzer-Platz 9, 8400 Winterthur, Switzerland; jessica.pehlke-milde@zhaw.ch; 2 Institute of Physiotherapy, School of Health Sciences, ZHAW Zurich University of Applied Sciences, Katharina-Sulzer-Platz 9, 8400 Winterthur, Switzerland; mandy.scheermesser@zhaw.ch; 3 Institute of Occupational Therapy, School of Health Sciences, ZHAW Zurich University of Applied Sciences, Katharina-Sulzer-Platz 9, 8400 Winterthur, Switzerland; martina.spiess@zhaw.ch (M.R.S.); christina.schulze@zhaw.ch (C.S.); 4 Institute of Nursing, School of Health Sciences, ZHAW Zurich University of Applied Sciences, Katharina-Sulzer-Platz 9, 8400 Winterthur, Switzerland; daniela.haendler-schuster@zhaw.ch; 5 UMIT TIROL Institute for Nursing Science, Private University of Health Sciences and Health Technology, 6060 Hall in Tirol, Austria; 6 School of Nursing, Midwifery, and Health Practice, Faculty of Health, Victoria University of Wellington, Wellington 6012, New Zealand

**Keywords:** migration, minority, digital health, participatory research, health technology

## Abstract

Digital health interventions (DHIs) are increasingly used to address the health of migrants and ethnic minorities, some of whom have reduced access to health services and worse health outcomes than majority populations. This study aims to give an overview of digital health interventions developed for ethnic or cultural minority and migrant populations, the health problems they address, their effectiveness at the individual level and the degree of participation of target populations during development. We used the methodological approach of the scoping review outlined by Tricco. We found a total of 2248 studies, of which 57 were included, mostly using mobile health technologies, followed by websites, informational videos, text messages and telehealth. Most interventions focused on illness self-management, mental health and wellbeing, followed by pregnancy and overall lifestyle habits. About half did not involve the target population in development and only a minority involved them consistently. The studies we found indicate that the increased involvement of the target population in the development of digital health tools leads to a greater acceptance of their use.

## 1. Introduction

Digital technologies such as mobile apps, text messaging and wearable devices represent an increasingly large field in health, promising to deliver individualized health care to a wide public. Numerous studies, however, have shown that digital health care is not equally distributed and disadvantages already vulnerable groups, thus sustaining or increasing existing health disparities [1,2,3,4,5]. Initial studies analyzing equity within digital interventions looked at whether disadvantaged groups such as minorities or migrants are less likely to use digital interventions. They found that smartphone and digital device use is also widespread among these groups [6,7] and that there is increasing openness to the use of digital health interventions and health information seeking using mobile devices [6,8]. However, studies have shown that health apps are rarely tailored to disadvantaged communities, although the degree of community-engagement in the development of health apps is key for usability and adherence [9].

In technology development, the involvement of potential users plays an important role. To implement this, there are various models, for example user-centered design (UCD) [10] and participatory design (PD) [11]. UCD starts from the needs of the users and involves them throughout the technology development process. PD starts earlier than UCD and collects user needs before product development. In addition to this, PD focuses on “human action and people’s rights to participate in the shaping of the worlds in which they act”, with participation focusing on “the fundamental transcendence of the users’ role from being merely informants to being legitimate and acknowledged participants in the design process” (pp. 4–5 [11]). End users can create content and make decisions (about features and appearance). In contrast to UCD, PD views potential users as partners and involves them in the entire technology development process. Therefore, in this scoping review we will be focusing closely on migrants and ethnic or cultural minorities and the development of Digital Health Interventions (DHI) for and with these communities. We use the term DHIs to refer to digital technology that is applied to achieve health goals [12]. Their range is broad and can reach from electronic medical records to mobile health apps used [13]. We will be looking at the level of participation or co-design, in the development of digital interventions and how it influences the outcomes, usability and adherence of the DHIs. Our hypothesis is that higher participation will lead to a more nuanced and targeted design, as well as to better health outcomes and more widespread use of the digital intervention.

In this study, we are using Lai et al.’s (p. 758 [14]) definition of migrants “as persons residing in a country who were born outside of that country and who arrived through an immigration or refugee program” [14]. Ethnic or cultural minorities are defined as persons which constitute “less than half of the population of the entire territory of a state whose members share common characteristics of culture, religion of language, or a combination of any of these.” [15]. Minority status is different from migrant or citizenship status as members of ethnic or cultural minorities can be citizens or non-citizens [14]. Furthermore, ethnic or cultural minorities include White, Black, Latino, Indigenous, Asian, Middle-Eastern and other minorities [16]. These groups are not homogenous, but face “similar challenges in terms of integration (…) related to discrimination, health status, civic engagement, and employment” (pp. 63–66 [14]). The terms “ethnic” and “cultural” minority are often used interchangeably in studies, sometimes in combination (“ethno-cultural” minorities). We will consistently be using both terms.

In this scoping review we want to analyze which digital health tools have been developed specifically for this diverse population, what health conditions or aspects of wellbeing they tackle and what the outcomes of their use are. We particularly aimed to map the degree of participation of the target groups in the identified studies and discuss its implication for the successful uptake of digital health technologies and actual impact on health outcomes.

## 2. Materials and Methodology

**Figure 1:**
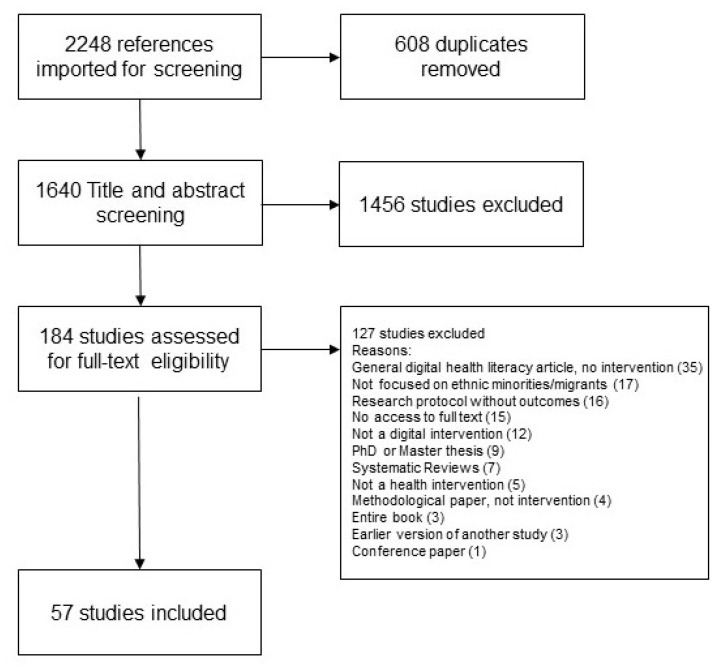
PRISMA flow diagram.

We chose the methodological approach of the scoping review because the literature in the field of digital health interventions for minority/migrant groups is very heterogenous. Scoping reviews are particularly useful to “identify main concepts, theories and knowledge gaps” (p. 467 [17]) in fields where few overview studies or systematic reviews exist. Our scoping review aims to provide a focus on the digital health interventions developed for minority and migrant groups, the health problems they address, their effectiveness at the individual level and the level of participation of the target groups in the development. We follow the methodology outlined by Tricco [17] and Arksey and O’Malley [18]. This means all steps and results of the search were documented and reported following the PRISMA Extension for Scoping reviews (PRIMSA-SCr) Checklist (see Figure 1).

### 2.1. Defining Relevant Terms

The initial keywords were digital tools, migration, user groups and health care. The search strategy using all identified key words can be found under the search term overview which was published at the Open Society Foundation (OSF) [19].

### 2.2. Searching and Identifying Relevant Studies

The studies were identified through a systematic search of Medline, PubMed, Cochrane, CINAHL and Google Scholar in January 2021. Reference lists of the chosen sources were reviewed to identify any further relevant sources. We restricted our search to articles published after 2007 as smartphones and mobile apps were rarely available before.

The initial search yielded 2248 potentially relevant citations. Five authors (I.R., M.S., M.R.S., C.S. and D.H.) reviewed titles and abstracts to determine relevance for further review of the full texts in accordance with inclusion criteria. The inclusion criteria for the title and abstract screening were the following:

1) The target groups of the study are ethnic or cultural minorities or migrants,
2) Digital health tools are used or developed as an intervention,
3) The intervention targets specific or general health issues (including wellbeing and health literacy).

All three aspects needed to be present for a publication to be included. Publications that did not contain all three criteria were excluded.

### 2.3. Study Selection

To manage the independently reviewed title and abstract screening process, we used Covidence Software (Covidence Systematic Review Software, Veritas Health Innovation, Melbourne, VI, Australia). We imported 2248 studies into Covidence, where duplicates were automatically signaled and removed based on an exact match of the title, date and author (608 duplicates). Two reviewers independently reviewed each title and abstract and conflicts were resolved by a third reviewer.

To ensure a common understanding of the inclusion criteria, reviewers met several times and discussed “conflict” cases, where the ratings of studies did not match. After the end of the title and abstract screening, a second round of title and abstract screening was done, sending all papers back into screening, as we felt that our understanding of the research question and inclusion criteria had become clearer and more aligned. The PRISMA diagram below reflects this second round of title and abstract screening, where 1456 studies were ultimately excluded (see Figure 1).

We then included 184 studies in the full-text screening. During this stage, two reviewers read the full text versions of the papers and a third reviewer decided in case of conflict. Additionally to the already defined inclusion and exclusion criteria (see above), several exclusion criteria were added (see Figure 1). Thus, studies were excluded if they were general articles on the digital health literacy of migrant populations, which did not define or test digital interventions (35), if they were not sufficiently focused on ethnic minorities or migrants (17), if they were research protocols without outcomes where digital interventions are presented without delving into use and viability (16), if they did not include digital interventions (12) or if they were not health interventions (5). Furthermore, several papers were excluded for formal reasons: because we had no access to the full text version (15) or because they were PhD or master theses (9), reviews (systematic, scoping, narrative, etc.) (7), entire books (3), methodological (4) or conference papers (1). Lastly, we excluded papers that were earlier versions of studies we included in our scoping review (3).

Before extraction we proceeded with the reference screening of the studies. A manual search of the reference lists of all included articles (title and abstract, then full text screening by the three main authors) resulted in an additional three studies which were included in the review.

## 3. Results

A total of 57 publications that met all inclusion and exclusion criteria were included in the final review.

### 3.1. Characteristics of Included Studies

Table 1 summarizes the characteristics of the included studies. We charted the studies according to region/country of provenance, study design, number of participants, target population, technology, area of intervention, health/wellbeing problem and degree of participation of target groups.

### 3.2. Region

Among the 57 studies included in our final selection, 37 (65%) were from the USA, nine were from countries of the European Union (16%), four (7%) were from Australia/Oceania, two (4%) were from Asia, two (4%) were from Canada, one (2%) was from the UK and one (2%) was from Africa. One (2%) study was a collaboration between Germany, Sweden and Egypt and another (2%) was a collaboration between Germany, Austria, Switzerland and Liechtenstein.

### 3.3. Study Design and Number of Participants

A total of 19 studies (33%) were randomized controlled trials (RCT), 15 studies (26%) used qualitative methods and seven (12%) used mixed methods (qualitative and quantitative methods). Two studies (4%) were non-randomized studies and four (7%) were cohort studies. Two (4%) were feasibility and acceptability studies and two (4%) were cross-sectional studies. Two (4%) were single-group pre- and post-test studies, one (2%) was a feasibility and acceptability study, one (2%) was an experimental study and one (2%) was an intervention study. The number of participants ranged from 9 [43] to 1512 [48].

### 3.4. Target Population

Among our studies, 46 out of 57 (81%) focused on migrants or ethnic minorities as the target population, while seven (12%) focused on refugees or displaced persons. Four studies (7%) focused on a combination of migrants, ethnic minorities and refugees, ethnic minorities and sexual minorities, as well as low-income individuals and migrants and ethnic minorities.

### 3.5. Digital Technology

Seven different types of digital technologies were identified that were used or developed in the studies: 20 mHealth/Apps (35%), 13 websites and/or informational videos (22%), 8 studies (14%) using text messages, 8 studies (14%) using other telehealth-technologies and 4 studies (7%) using technologies from a combination of websites, email, apps and text messages. Two studies (4%) focused on social media and a further two studies (4%) used other technologies such as interactive assessments and YouTube.

### 3.6. Intended Use of the Digital Tools

We found that the digital tools developed, tested or evaluated in the publications were aimed at the following eight areas of health intervention (as declared in the respective publications) in decreasing order of representation in our sample:

- Providing *health information* to a population afflicted by a particular illness or condition (22 studies, 39%).
- *Self-management* of illness, for instance, through the rating and tracking of symptoms and medication (14 studies, 25%).
- *Prevention* of illness (e.g., diabetes, weight gain) (seven studies, 12%).
- Facilitating *consultations* with health professionals either through reminders for appointments or through e-consultations (six studies, 10%).
- Facilitating *patient support groups* or *patient networking* (three studies, 5%).
- *Hybrid* forms of intervention, comprising of two or more of these dimensions (three studies, 5%).
- *Language translation,* usually during consultations (one study, 2%).
- *Home monitoring system* to track movement (one study, 2%).

### 3.7. Health Focus

The selected studies addressed various health and wellbeing topics and risks and illnesses in decreasing order: mental health/wellbeing (14 studies, 25%), pregnancy and/or postpartum (eight studies, 14%), overall health–lifestyle-habits (eight studies, 14%), HIV and/or other sexually transmitted diseases (STDs) (eight studies, 14%), diabetes (five studies, 9%), infectious diseases (four studies, 7%), cancer (four studies, 7%), obesity (three studies, 5%), neurological conditions (two studies, 3%) and cardiac diseases (one study, 2%).

### 3.8. Degree of Participation of Target Groups in DHI Development

Our most important unit of analysis was the degree of participation of the target groups. Some studies did not ask the target groups for any feedback or involve them in any way in the development of the interventions, while others involved these stakeholders in every step of the process.

**Figure 2:**
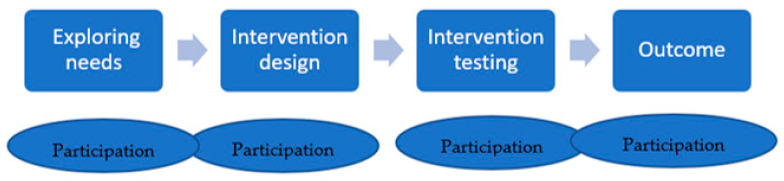
Phases of DHI development.

Based on the participation of the target groups during the development of the DHIs described in our studies, we outlined four phases of DHI development inductively (see Figure 1) and developed a typology of our studies according to the degree of user involvement and the timing of participation (Figure 2).

We identified four types of studies:

**Type 1**: Participation at the outcome stage of intervention;
**Type 2**: Participation before intervention design;
**Type 3**: Participation in intervention testing;
**Type 4**: Participation throughout the development process.

#### 3.8.1. Type 1: Participation at the Outcome Stage of Intervention

Among the selected studies, 33 out of 57 (55%) can be categorized as type 1, with the least amount of participation of the target groups in any phase of development of the digital health intervention. These studies usually investigate the effectiveness of the interventions in terms of outcomes. It is at this final stage that participants are asked to provide feedback on efficacy, acceptability or usability. The acceptability, feasibility and usability of the intervention are often evaluated through a secondary data analysis (e.g., length of use of the DHIs). Sometimes acceptability is analyzed through short questionnaires (yes/no answers or open answers) about the usefulness of the intervention [36]. Rarely, more in-depth feedback is provided in qualitative interviews [43] or focus groups [48]. This feedback is not considered for an adaptation of the interventions, though it is not excluded that this might be done in the future. Most of the studies in this category are RCTs with a minority of qualitative or mixed-method studies. The most frequently used digital interventions were apps, websites, text messaging and telemedicine. Many of these studies address the health issue of mental health and well-being, as well as overall lifestyle issues.

Often these are digital interventions that have been developed for the general population, that are now being used for more ethnic and culturally diverse populations with few or no adaptations.

Some studies show a short-term improvement of illness symptoms such as depression or other mental health issues [26,42,52], diabetes [27], a better knowledge of health information [20,30,44], increased mammogram take-up [32] and an increase in positive health behaviors, such as vaccination [29,31]. Some of these DHIs show a good acceptance among the target population [23,26,35,36,39]. However, not all interventions are successful, with some studies reporting participant resistance to digital solutions and a preference for in-person care [22,40,45], a tendency to worsen the exclusion of less tech-affine individuals [37], no positive effects [46] or a drop in positive effects of the DHIs once more time has passed and adherence has dropped [25].

Lee et al. [32] showed that digital interventions can reach underserved and hard-to-recruit populations that bear disproportionate cancer burdens. The study by Borsari [23] showed a high acceptance towards the Pregnancy and Newborn Diagnostic System (PANDA) for antenatal care for a multiethnic and mobile population. Studies in which text messages are used seem to be effective and help improve health and wellbeing outcomes. For example, messages improved women’s mood, helped them feel more connected with their social environment [26], engaged patients in their health and increased the rate of influenza vaccinations [29,31]. Röhr et al. [40] were further able to show a high usability of the Sanadak digital intervention (SUS-usability score of 78.9 within a range of 0 to 100). It aimed at reducing mild-to-moderate post-traumatic stress in Syrian refugees. However, this intervention was not more effective than the control condition and not more cost-effective. Therefore, the authors found that Sanadak is not suitable as a stand-alone treatment.

#### 3.8.2. Type 2: Participation before Design

Type 2 is characterized by exploring the needs of the target population before developing the intervention. This is done to better tailor the intervention to the specific needs of the target group. Eight studies (12%) can be classified as this type. Four of the eight studies developed a digital intervention: An SMS program of information for pregnant African American and African Caribbean immigrant women in New York City [53], a personal digital assistant program to promote fruit and vegetable intake to low-income, ethnic minority girls [57], websites, including YouTube, about HIV/STDs for ethnic, racial, sexual minority 15–24-year-old adolescents [60] and a culturally sensitive technology-based campaign focused on HIV testing [60]. One study used a Consumer Assessment of Healthcare Providers and Systems (CAHPS) of the need to adapt interventions: e.g., a telehealth service for Colorado Young Adults with Type 1 Diabetes (T1D) was adapted for young adults from racial/ethnic minorities and low socioeconomic backgrounds with T1D [58]. Another study used text messages to provide diabetic retinopathy awareness and improve diabetic-eye-care behavior for indigenous women with or at risk of diabetes [59]. One study examined the use of a digital intervention, text messages called mMom, to improve access to maternal, newborn and child health service for ethnic minority women in Vietnam [56] and one study explored health education videos for acceptability by Somali refugee women [54].

To engage the target population and understand participants’ perceptions of the use and needs before developing the intervention, four of seven studies used a mixed method approach. Blackwell et al. [53] conducted focus groups and used key informants, interviews and observations. The resulting qualitative themes were used to develop a survey instrument. Nollen et al. [57] employed focus groups and a Health Technology Questionnaire. Raymond et al. [58] employed a patient advisory board, stakeholder focus groups and a survey. Umaefulam et al. [59] utilized Sharing Circles and a survey.

Five studies used a participatory approach to investigate the needs of the target groups: McBride et al. [56] used various participatory methods including focus groups [54], ethnography and interviews and Whitley et al. [60] used focus groups. Raymond et al. [58] involved Patient Advisory Council members in the project to discuss their experiences, preferences and priorities for telehealth care for diabetic patients and their interest in participating in group appointments. Umaefulam et al. [59] used Sharing Circles to gain an inside perspective from indigenous women involved in the intervention to increase the cultural sensitivity in the developing content for the intervention.

Nollen et al. [57] were able to show that the iterative involvement of young people in all phases of the development of the wearable computer program was successful in creating changes in participants’ health behaviors. Umaefulam et al. [59] were able to show that mHealth education increased awareness and resulted in a change in diabetic-eye-care behaviors.

#### 3.8.3. Type 3: Participation in Intervention Testing

Type 3 is characterized by the involvement of the target group during the testing phase and an adaptation of the intervention. Nine of the fifty-seven studies (17%) can be classified as this type. In five of the ten studies, the specific needs of the target group were first identified. Then, the DHIs were adapted according to these needs.

As in type two, the involvement of the target group in type three was carried out using qualitative methods in most of the included studies. Seven of the ten studies used qualitative methods: Handley et al. [63] and Lee et al. [32] conducted focus groups, Burchert et al. [61], Liss et al. [65] and Quarells et al. [67] conducted both focus groups and interviews and Zheng and Woo [52] assembled the participants for a talking-based workshop. The other three studies used quantitative methods: Muroff et al. [66] used the collected data from an app, Tanner et al. [69] carried out a survey and Liss et al. [65] and Dorfman et al. [63] used mixed-method approaches. In terms of digital tools, three studies used telehealth technologies, four used apps, one used videos, one used text messages, one used social media and one used YouTube videos.

The studies identified several advantages of using digital technologies. Dorfman et al. [62] and Lee et al. [64] were able to show an improved accessibility of underserved patient populations when technologies were adapted. The study of Dorfman et al. [62] suggests that appropriately adapted mobile-health technologies may provide an avenue to reach underserved patients and implement behavioral interventions to improve pain management. The study of Lee et al. [64] demonstrated how culturally relevant information can be collected to develop a text message intervention to incorporate the perspective of the target population at the intervention development stage. The study findings may help in the development of future interventions targeting different types of cancer screening in other underserved racial or ethnic groups.

Muroff et al. [66] and Quarells et al. [67] were able to show that digital interventions have the potential to expand access to culturally and linguistically competent services. In addition, Quarells et al. [67] adapted their project UPLIFT, a digital health intervention for the self-management of depression to Black and Hispanic people with epilepsy, through a careful and systematic adaptation process to new populations or cultural settings. This shows that digital interventions can expand the available strategies of a health problem while needing to be carefully and systematically adapted to the new population or cultural setting.

#### 3.8.4. Type 4: Participation throughout Development Process

Type 4 represents the kind of studies that take the tailoring of the digital intervention to the target group in question one step further. A digital intervention is thus not merely adapted to a new group of participants, but the intervention is developed from its inception according to the needs of the group and with their participation at several stages of development and/or implementation. It is the most participative type of DHI and the closest to a co-design process [70,74,75]. Only seven of our studies correspond to this type with the highest degree of participation (12%). Most studies in this category are qualitative studies, using qualitative interviews, focus groups and mixed methods. One study [72] is an RCT and one [70] combines a RCT and a focus group.

These studies report a high level of success in changing health behaviors, improving health knowledge, lowering barriers, as well as improving acceptability, usability and satisfaction. They focus on understanding the needs of the target group before developing the digital technology. To comprehend the participants’ needs, the studies used mainly qualitative methods, such as focus groups, to understand women’s views of breast cancer, attitudes toward mammography and preferred content, or meetings with community health leaders involved in cancer care and women’s health [76]. The focus groups found that a community-based, participatory social marketing approach can be used successfully to create more culturally appropriate text messages in order to lower barriers for HIV testing [73].

Wang et al. [76] concluded that integrating dermatology care through a telemedicine system can lead to improved access for underserved patients. The results show that tele-dermatology is increasing in adoption for diverse patient populations. This is evidenced by the higher number of underserved and underinsured groups that were reached with tele-dermatology compared to conventional referrals, providing a much-needed service to a more diverse patient population. In addition, fewer appointments were missed and users were able to see a dermatologist in person and receive skin cancer treatment more quickly compared to conventional referrals. Brewer et al. [70] developed a culturally relevant cardiovascular health and wellness mobile health tool using a community-based participatory approach. The result was that culturally relevant lifestyle interventions could be implemented and delivered by mobile health tools.

## 4. Discussion

### 4.1. Principal Findings

This scoping review aimed to analyze which digital health tools are available for migrant and cultural or ethnic minority populations, as well as what role their participation plays in the development and successful uptake of DHIs. Since new technologies such as apps or smartphones have only been available since ca. 2007, studies focusing on DHIs developed for immigrant, ethnic and cultural minorities are also relatively new.

Our study highlights the importance of the use of participatory methods in the development of DHIs for migrant and cultural or ethnic minority populations. We identified four types of DHIs describing different degrees of target group participation in the development of digital intervention, with Type 1 representing the lowest level of participation and Type 4 the highest. We did not use existing models of digital health intervention development. Instead, we inductively analyzed the stages of development as well as the degree of participation of the target groups, as described in the studies we included in our review.

Overall, there seems to be a larger focus on developing DHIs for ethnic or cultural minorities and migrants in the USA (e.g., Latino or Black communities), where 65% of our 57 studies were from, and less on minority and migrant communities in Europe, Asia, Africa and Australia. While findings from different regions can be transferred, addressing this unequal distribution in the future is important as population diversity and heterogeneity are global phenomena. Furthermore, the studies we reviewed focused mostly on migrants and cultural or ethnic minorities and less on refugees, showing a general tendency to prioritize the development of DHIs for people with a settled legal status over those with a precarious or unclear immigration status.

In terms of methodology, we found that the most common types of studies were RCTs (34%) followed by qualitative studies (30%). This is an interesting development as qualitative or mixed-method studies especially allow the use of participatory methods, showing a growing concern for this topic. The most widely used technologies were mhealth interventions (35%), followed by websites and informational videos (23%), text messages (14%) and telehealth (14%). Indeed, many of the studies we reviewed highlighted the increasing importance of mobile phones in providing low-threshold health interventions for marginalized populations, as well as for the delivery of tailored health information in order to motivate, provide support and empower individuals.

Among the studies we reviewed, most were aimed at illness self-management, followed by consultations and prevention. The main health issues the DHIs addressed were mental health and wellbeing (23%), followed by pregnancy and postpartum (17%) and overall lifestyle habits (15%), which correspond to some of the leading health challenges that migrant and minority populations are faced with over time in host countries [77,78].

Most of the studies we found (53%, N = 28) are type 1, meaning that their digital intervention is evaluated in terms of acceptability with the target population, feasibility or effectiveness after it has been developed. There is typically no feedback loop allowing for an adaptation of the digital interventions according to the findings of the studies or feedback from the target group. Type 2 (11% of studies) includes a higher degree of participation than type 1, as the needs of the target population are identified before developing the intervention. Type 3 are studies where the target group participated during the testing phase of the digital intervention as well as in the adaptation of the intervention according to the community needs. This was the second most common type of study (23%). Type 4 (11%) represented the most inclusive studies where the target group was involved in every step of development and evaluation from analyzing their needs to the development, testing and use of the intervention.

Studies classified as type 2, 3 and 4 used more qualitative methods than studies in type 1. Our review indicates that focus groups, interviews and mixed-method approaches are particularly suited for a community health approach to DHI development.

Overall, the studies in our review show the importance of language in the development of DHIs in order to reduce barriers to the uptake and use of digital health tools [79]. Thus many of the analyzed DHIs included health information not just in the national language, but also in the native languages of target populations. Some went a step further and culturally tailored the messaging to the communities in question. This type of tailored development seems to increase the ability to reach particularly underserved groups, yet it also requires a higher degree of target population involvement and co-design. This echoes findings from other studies such as Gonzalez et al. [80], which showed that participatory design and co-design can benefit long-term engagement with mHealth tools, and Jang et al. [81], who showed how participation can improve access and enrollment in digital interventions. However, as analyzed by Evans et al. [82], in future work, the definitions of participatory design or co-design need to continue to be scrutinized in order to better understand and improve the impact of these design practices on equity in health.

### 4.2. Limitations

One of the limitations of our study lies in the definition of migration and ethnic or cultural minorities that we used for our search. This concept includes populations who are White as well as People of Color, migrants as well as refugees, and we found them to be some of the most widely used concepts in health research internationally. We did not, however, include similar concepts such as “other” or “othering” that are more widely used in social sciences and migrations research. This could have potentially yielded further search results. Similar to Gonzales [80], we considered the effectiveness of the digital interventions at the individual level based on the authors’ self-reports. Additionally, though we presented the impact DHIs had on health and wellbeing outcomes as well as long-term usability and adherence to treatment or lifestyle changes, our insights are limited by the time horizon of the studies. In the future, an updated scoping review or other systematic reviews should be conducted to measure the long-term impact of participatory approaches. It may also be that studies were conducted at various stages of technology development or follow-up studies were conducted but not published. Furthermore, we must be aware that negative or not suitable results are usually not published. The authors of the study have different disciplinary backgrounds, including nursing, physiotherapy, occupational therapy and sociology. In this way, they bring in different perspectives that may have led to different ratings of the studies. As with all other interventions, it is an open question whether these effects last in the long term.

## 5. Conclusions

The previous literature has pointed out the importance of participatory and user-centered development of digital health interventions and the circular involvement of end users, especially to reach communities that face particularly high barriers to healthcare [80]. Migrants, refugees and cultural or ethnic minorities are among these particularly vulnerable groups. In our scoping review, we focused on the digital health interventions aimed specifically at these groups and analyzed their self-reported effectiveness, as well as the degree of participation of their target group, in the development or implementation of the intervention. We found that despite the high health needs of this population, among 2248 studies, only 57 targeted migrants, refugees or cultural or ethnic minorities in particular. Of these 57 studies, about half applied the digital health technology developed for the general population to the migrant or minority populations without making any adaptations to their specific cultural, linguistic or health needs. The other half assessed their needs, adapted the digital interventions accordingly and/or involved the target population in development and/or testing. Only a small fraction of the studies included in this review reported a high level of participation of migrant and cultural or ethnic minority populations in the design of the digital health tools. This indicates that such practices continue to be the exception rather than the norm in DHI development. Findings from these studies, however, seem to indicate that increased participation has the potential to improve health outcomes, acceptance and use of DHIs in migrant and minority populations.

## Figures and Tables

**Table 1:** Characteristics of the studies.

Reference	Aim	Target Population and Region	Study Design	Participation of Target Group	Health Focus	Digital Technology	Outcome
**Type 1: Participation at the outcome stage of intervention (33 studies)**							
Abu-Saad et al. [20]	Develop an Interactive Lifestyle Assessment, Counseling and Education (I-ACE) software to support dietician-delivered lifestyle counseling among the target group.	50 overweight or obese Arab adults (aged 40–62 years)	RCT	4 in-person, dietician-delivered counseling sessions over 6 months using the I-ACE.	Diabetes	Interactive Lifestyle Assessment	The culturally adapted software for dietician-delivered lifestyle counseling could increase the pace of acquiring diabetes-related lifestyle knowledge and showed a trend toward improving lifestyle behaviors.
Bender et al. [21]	Assess feasibility and potential efficacy of a culturally adapted mHealth weight loss lifestyle intervention.	Overweight Filipino Americans with type 2 diabetes (T2D).	RCT	45 Filipino American adults were enrolled and randomized.	Obesity	mHealth/ App	The PilAm Go4Health was feasible and demonstrated potential efficacy in reducing diabetes risks in overweight Filipino Americans with T2D.
Berridge et al. [22]	Examine the experience of low-income immigrant senior residents’ family contacts and staff with remote monitoring technology.	Minority seniors (different origins) in USA	Qualitative	Experience with technology discussed in qualitative interviews.	Overall lifestyle habits	Telehealth	Resistance to use of remote monitoring technology rooted in cultural expectations of care.
Borsari et al. [23]	Adapt the Pregnancy and Newborn Diagnostic System (PANDA) for antenatal care for a multiethnic and mobile population.	Women Asylum seekers in Italy	Feasibility study	Time spent for each visit (indirect measure), yes/no questions, questionnaire for feedback.	Pregnancy and/or postpartum	mHealth/ App	The study noted a high patient satisfaction rate (91.9%). The system was efficient in providing comprehensive and high-quality antenatal care amongst migrants, facilitating the continuity of care for a population undergoing frequent relocations.
Chiu et al. [24]	Assess the usability of a new Internet-based Caregiver Support Service and evaluate its effects on health outcomes.	Chinese Canadians who care for a family member with dementia	Mixed Methods	Demographic and questionnaire data were collected from 28 participants, and in-depth interviews were conducted with 10 participants.	Neurological conditions	Asynchronous e-mails and website	This study showed that caregivers can benefit from receiving professional support via asynchronous e-mails and a special information website. The Model is a feasible approach for supporting caregivers who prefer an alternative service model.
Comulada et al. [25]	Examine CEMA (cell phone-based ecological momentary assessment) and PFR (photographic food records) adherence to the use of a mobile app designed to help mothers self-monitor lifestyle behaviors and stress.	Ethnic minority mothers in USA	RCT	Selection of measures tailored with focus groups before RCT	Pregnancy and/or postpartum	mHealth/ App	Variations in population and temporal characteristics should be considered for mobile assessment schedules. Neither CEMA nor PFR alone is ideal over extended periods.
Garcia et al. [26]	Design and testing of a remote psychosocial therapy system to improve personal health via text messaging; (2) reflection on the usability and feasibility of mobile phones in psychosocial therapies.	Immigrant women claiming social assistance who use social services in Spain	Non-randomized experimental study	Women interviewed before study to reach a psychological diagnosis. Intervention compared face-to-face therapy session and text messages to face-to-face therapy and no text messages. Pre-defined message banks in Spanish are used. Women are not involved in their creation.	Mental health and wellbeing	Text messages	Messages improved women’s mood and helped them feel more connected with social environment. Acceptability was good.
Hu et al. [27]	Examine the feasibility and acceptability and pilot test the potential efficacy of a social media-based DSME intervention among low-income Chinese immigrants with type 2 diabetes (T2D) in New York City.	Low-income Chinese immigrants in New York City	Pre- and post-test	Acceptability was assessed via a satisfaction survey at 3 months.	Diabetes	Videos and social media	The results of this study demonstrated high feasibility and acceptability of this intervention. The retention rate was comparable to or better than that previously reported in in-person diabetes interventions with Chinese immigrants.
Johnson et al. [28]	Explore midwives’ and non-Western immigrant women’s attitudes towards and experiences of using the MAMAACT intervention.	Midwives and Non-Western immigrant women in European Union	Qualitative	Focus groups with the midwives, semi-structured interviews with the women, observation of both during consultations. Participatory approach with the midwives, but not the women. Women’s accounts only included after instrument development.	Pregnancy and/or postpartum	mHealth/ Apps	MAMAACT intervention is a tool to build knowledge and skills’ and ‘intervention experiences. Sessions promoted midwives’ reflection on practice; however, at the visits, habitual ways of interacting impacted encounters between midwives and non-Western immigrant women.
Kharbanda et al. [29]	Evaluate targeted text message reminders for low-income, urban parents to promote influenza vaccination among children and adolescents.	Low-income, urban population, 88% publicly insured and 58% from Spanish-speaking families.	RCT	9213 children and adolescents aged 6 months to 18 years receiving care at 4 community-based clinics.	Infectious diseases	Text message	Compared with usual care, the text messaging intervention was associated with an increased rate of influenza vaccination.
Kiropoulos et al. [30]	Investigate the effects of multicultural information on depression literacy, depression stigma, depressive symptoms.	Greek- and Italian-born immigrants in Australia	RCT	Follow-up questionnaire about symptoms. No feedback on intervention.	Mental health and wellbeing	Website and/or informational videos	Significant differences between the intervention group and the control group were reported for depression literacy and personal stigma scores, but not for perceived stigma or level of depression scores.
Kumar et al. [31]	Assessed whether patients would be receptive to influenza vaccination text messages.	Low-income, racial and ethnic minority primary care patients	Non-randomized experimental study	Participants completed a self-administered survey after the intervention.	Infectious diseases	Text message	Text messaging is a feasible tool to engage patients in their health and improve annual influenza vaccination rates among low-income, racial and ethnic minority patients.
Lee et al. [32]	Explore the impact of mMammogram on changes to study participants’ screening behavior and suggest how the intervention can be improved for wide dissemination and implementation in the Korean American community.	Korean American immigrant women	Qualitative	Focus groups with Korean immigrant women who completed the mMammogram.	Cancer	mHealth/ App	A mobile app intervention that is culturally tailored, along with health navigation services, can be a feasible, effective and acceptable tool to promote breast cancer screening behaviors in underserved immigrant women.
Linke et al. [33]	Examine participant engagement of an internet-based physical activity (PA) intervention for Latinas, adoption and maintenance of PA behavior change.	Latina women, USA	RCT	None.	Overall lifestyle/health	Website and/or informational videos	The use of a tailored, web-based PA intervention was significantly related to increased PA levels in Latinas.
Li et al. [34]	Test the feasibility of a community health worker-led, mHealth-based diabetes self-management education and support (DSMES) intervention to reduce disparities in accessing DSMES in the target group.	Latinos living in rural South Texas	Pre- and post-test	Patient satisfaction and acceptance by questionnaire.	Diabetes	mHealth/ App	A community health worker-led mHealth-based intervention was feasible and acceptable to improve access to DSMES services for Latino adults living in rural communities.
Liu et al. [35]	Test app usability and acceptability for Chinese immigrant caregivers.	Chinese-speaking caregivers	Qualitative	In-lab testing and at-home testing with interviews (face-to-face and phone interviews) for user experience, usability and acceptability.	Overall lifestyle/health	mHealth/ App	Positive overall experience, more in-depth feedback planned in future studies.
Müller et al. [36]	Design and development of a digital communication assistance tool (DCAT) for 19 different languages and dialects for collecting patients’ health complaints and medical history.	Refugee patients, Germany	Qualitative	Data analysis of app use and a questionnaire on whether patients were able to use DCAT well.	Overall lifestyle/health	mHealth/ App	High acceptance and usability of the app by patients. Using digital tools for overcoming language barriers can be a feasible approach when providing health care to foreign-language patients.
Nelson et al. [37]	Design a secure messaging service (SMS) and Interactive Voice Response (IVR)-delivered mHealth intervention to improve medication adherence among low SES, diverse adults with type 2 diabetes (T2DM), called the MED intervention.	Diverse adults with low SES	Intervention study	80 patients with T2DM participated in a 3-month mHealth intervention called Messaging for Diabetes that leveraged a mobile communications platform.	Diabetes	mHealth/ App	Racial/ethnic minorities, older adults and persons with lower health literacy or more depressive symptoms appeared to be the least engaged in intervention.
Nollen et al. [38]	Test a 12-week mobile technology intervention as a stand-alone tool to improve diet habits.	Racial/ethnic-minority girls	RCT	Girls randomized to the control condition received modules at Weeks 1–4 (fruits and vegetables), Weeks 5–8 (sugar-sweetened beverages) and 9–12 (screen time). No feedback from participants was provided.	Obesity	mHealth/ App	A stand-alone mobile app may produce small-to-moderate effects for fruits/vegetable consumption and reduced sugar-sweetened beverage consumption.
Perrino et al. [39]	Participation and prediction of participation in eHealth Familias Unidas intervention, aimed at reducing substance use and other health risks among Hispanic youth.	Hispanic families with high-risk youth in USA	RCT	No feedback from participants, only secondary data analysis of the eHealth intervention.	Overall lifestyle/health	Website and/or informational videos	Participation in eHealth intervention was higher than in face-to-face intervention. This suggests that Internet-delivered, family-based interventions are a feasible and promising method for reaching Hispanic families with high-risk youth.
Röhr et al. [40]	Evaluate effectiveness and cost-effectiveness of Sanadak-app, an app for treating post-traumatic stress syndrome.	Syrian refugees, Germany	RCT	No feedback concerning the app, only analysis of mental health scores and symptoms after each phase (effectiveness).	Mental health and wellbeing	mHealth/ App	Sanadak was not more effective in reducing mild-to-moderate post-traumatic stress in Syrian refugees than the control condition, nor was it likely to be cost-effective. Therefore, Sanadak is not suitable as a standalone treatment. However, as the app usability was very good, no harms were detected and stigma significantly reduced, Sanadak has potential as a bridging aid within a stepped and collaborative care approach.
Schulz et al. [41]	Assessed the demographic and disease profile of refugee patients attending a telehealth clinic, calculated patient travel avoided. Assessed challenges and performance of two videoconferencing solutions.	Refugees/displaced persons/asylum seekers in Australia	Cohort study	No feedback from participants provided.	Infectious diseases	Telehealth	Effective in terms of avoiding travel and CO_2_ production. Technical issues in 25% of consultations. A stronger internet connection was needed to ensure quality of videoconference.
Spanhel et al. [42]	Examined feasibility, acceptance and preliminary effectiveness of a culturally adapted digital sleep intervention for refugees.	Refugees	RCT	Baseline assessment (T1), follow-up assessments took place 1 month (T2) and 3 months after randomization (T3).	Mental health and wellbeing	Website and/or informational videos	Low-threshold, viable access to mental healthcare can be offered to multiple burdened refugees by culturally adapting an intervention, providing it in a scalable format and addressing transdiagnostic symptom, such as sleep disturbances.
Tanner et al. [43]	To develop and implement weCare, a bilingual mHealth intervention to support HIV care engagement among racially/ethnically diverse young GBMSM and transgender women.	Racially diverse gay, bisexual and other men who have sex with men (GBMSM) and transgender women	Cohort study	Interviews with intervention participants and HIV clinic providers and staff.	HIV and/or other STDs	mHealth/ App	Informed messages targeted to GBMSM and transgender women using tailored bidirectional messaging from a “real” person with whom participants have a relationship and personalized to participants. Outcomes become essential to ensure engagement in care and support health outcomes.
Thompson et. al. [44]	Investigate whether educational modules online increase immediate nutrition and feeding knowledge in low-income Spanish-speaking Latino immigrant parents.	Low-income Spanish-speaking latino immigrant parents, USA	RCT	No feedback from participants provided. Evaluation of knowledge change after use of intervention.	Overall lifestyle/health	Website and/or informational videos	Immediate parental knowledge was enhanced.
Tomita et al. [45]	Feasibility of depression screening by SMS among refugees in South Africa who attend mental health services.	Refugees, South Africa	Cohort study	Analysis of statistics and feedback to SMS-based intervention (overall evaluation, interest in future use, comfort of use).	Mental health and wellbeing	Text messages	Intervention is feasible for assessment of depression symptoms, comfort of use rated high. Face-to-face preferred for consultations.
Ünül Ince et al. [46]	Evaluate effectiveness of a culturally sensitive self-help intervention for reducing depressive symptoms.	Turkish migrants, Netherlands	RCT	No feedback from participants provided. Data analysis of depression scores.	Mental health and wellbeing	Website and/or informational videos	No significant effect on reduction of depressive symptoms.
Van der Veen et al. [47]	Effectiveness of a culturally tailored internet intervention promoting Hepatitis B virus (HBV) screening in Turkish migrants.	Turkish migrants	RCT	Participation in an online intervention. No feedback from participants.	Infectious diseases	Website and/or informational videos	The study was not able to demonstrate the added value of behaviorally plus culturally tailored information on screening uptake.
Wollersheim et al. [48]	Improve the psychosocial health of and facilitate settlement by mobile phone-based peer support.	Nuer (southern Sudanese) women, Australia	Qualitative	Fifth and final sessions were focus groups to evaluate the intervention.	Mental health and wellbeing	mHealth/ App	Women’s cognitive social capital was increased through intervention, with potential of using technology to bridge health inequities in a marginalized group.
Ye et al. [49]	Establish a telepsychiatry services that connect Korean mental health patients in Georgia with psychiatrists in California.	Asian Americans, USA	Mixed methods	Upon completion of the program, 16 patients completed a questionnaire.	Mental health and wellbeing	Telehealth	Participants appreciated cultural sensitivity of intervention and interaction with provider, but technical issues affect the quality of clinical interaction.
Yeung et al. [50]	Evaluate the effectiveness of a telepsychiatry-based culturally sensitive collaborative treatment (T-CSCT) intervention to improve treatment outcomes for Chinese American immigrants with major depressive disorder (MDD).	Chinese American Immigrants, USA	RCT	Eligible patients were randomized to receive either T-CSCT or treatment as usual (TAU) for 6 months.	Mental health and wellbeing	Telehealth	T-CSCT is effective in improving treatment outcomes of Chinese immigrants with MDD.
Young et al. [51]	Examined whether and how an HIV-prevention diffusion-based intervention spread throughout participants’ online social networks and whether changes in social network ties were associated with increased HIV prevention and testing behaviors.	Racial/ethnic minority men who have sex with men (MSM)	RCT	112 participants received peer-delivered HIV (intervention) or general health (control) information over 12 weeks through closed Facebook groups.	HIV and/or other STDs	Social media	Among high-risk MSM, peer-led social media HIV prevention interventions can increase community cohesion.
Zheng and Woo [52]	Compare YouTube against traditional talk-based workshops in delivering dementia knowledge to the target group.	Chinese American population	Quantitative	Data collected from YouTube viewing data and talk-based workshop participants’ demographics were analyzed.	Mental health and wellbeing	YouTube	Internet-based websites such as YouTube are no substitute for dementia education, but for the target group and their health, it is promising. However, talk-based workshops are still more desired for dementia education.
**Type 2: Participation before design (8 Studies)**							
Blackwell et al. [53]	Develop an SMS program (T4B-SMS program) as a source of information and resources for prenatal care at an urban health center in New York City.	Pregnant African American and African Caribbean immigrant women, USA	Mixed methods	Focus groups, key informants, interviews and observations. Qualitative themes used to develop a survey instrument.	Pregnancy and/or postpartum	Text messages	Receiving prenatal health electronic messages through texting is a positive avenue to provide pregnant women in NYC with information. More research is needed with a larger population and direct modeling of testing of the theoretical constructs is needed to fully assess the perceived usefulness and relative advantage of T4B in this population.
DeStephano et al. [54]	Evaluate acceptability of video prenatal education in an obstetric clinic for Somali refugee women.	Somali refugee women, USA	Mixed methods	Focus groups were used to develop the videos.	Pregnancy and/or postpartum	Website/informational videos	Videos were acceptable to target group with a preference for videos in Somali. Increased interaction during appointments according to providers.
Lee et al. [55]	Address the need for enhanced access to HIV prevention for Latinx immigrant sexual minority men. Development and piloting of a culturally sensitive technology-based campaign focused on HIV testing and pre-exposure prophylaxis (PrEP) uptake.	Latinx immigrant sexual minority men, USA	Mixed Methods	3 focus groups with 15 Latinx immigrant sexual minority men were used to refine the HIV prevention content, which was then piloted on social media platforms.	HIV and other STD	Social Media	Culturally relevant social media and web-based outreach strategies that are informed and developed by the community can reach Latinx immigrant sexual minority men for HIV prevention.
McBride et al. [56]	Pilot and evaluation of a low-cost mobile health (mHealth) intervention called mMom utilizing behavior change communication (BCC) to improve access to maternal, newborn and child health (MNCH) services and health equity among the target group living in remote areas.	Ethnic minority women (EMW), Vietnam	Qualitative	A pre- and post-intervention survey was administered to all participants.	Pregnancy and/or postpartum	Text messages	The messages promoted increased contact between participants and health providers, which holds potential to address the marginalization of ethnic minority women from the health system.
Nollen et al. [57]	Develop a personal digital assistant (PDA) program to promote increased intake of fruits and vegetables (FV) to the target group.	Low-income, ethnic minority girls	Mixed Methods	Focus groups and Health Technology Questionnaire.	Obesity	mHealth/ App	The findings suggest that adolescent girls like and will use a device that prompts actions toward specified goals. Early engagement of youth in development allowed for redesign that enhanced usability and had an impact on short-term consumption.
Raymond et al. [58]	Adapt the CoYoT1 Clinic model to address the needs of a low socioeconomic status (SES), racially/ethnically diverse YA population with type 1 diabetes (T1D). Assess the effects on access to care, follow-up frequency, psychosocial outcomes and patient and provider satisfaction.	Young adults (YA) from racial/ethnic minorities and low socioeconomic backgrounds	Mixed Methods	Patient advisory board, focus groups, survey.	Diabetes	Telehealth	The original CoYoT1 Clinic model was successfully adapted to serve a low SES, publicly insured, racial/ethnic minority YA population with type 1-diabetes. Better management of psychological co-morbidities apparent.
Umaefulam and Premkumar [59]	Explore utilizing mobile health (mHealth) via text messages to provide diabetic retinopathy awareness and improve diabetic-eye-care behavior.	Indigenous women with or at risk of diabetes	Cohort study	Sharing circle and survey.	Diabetes	Text messages	The mHealth education intervention increased diabetic retinopathy awareness and fostered a change in diabetes-eye-care behavior. Health information via text messaging can motivate, provide support and empower individuals as well as prevent and manage chronic conditions and reduce the risk of complications.
Whiteley et al. [60]	Develop an HIV/STI Internet intervention from publicly available websites including YouTube. Material chosen for the study is relevant to minority youth and to the IMB model. The preliminary efficacy of the intervention was tested in a small, randomized controlled trial.	Ethnic, racial, sexual minority adolescents	RCT	18 ethnically and sexually diverse urban youth provided feedback on the relevance, comprehensibility and acceptability of selected sites and content in focus groups.	HIV and/or other STDs	Website and/or informational videos	Significant changes were found in measures of self-efficacy and the reduction in unprotected sex acts suggesting that this easily disseminated Internet content could result in changed attitudes and behavior.
**Type 3: Participation in intervention testing (9 Studies)**							
Burchert et al. [61]	Adaptation of existing web-based e-mental health intervention into mobile mental health tool.	Syrian refugees residing in different countries	Qualitative	Three phases of adaptation with interviews, expert interviews and focus groups. Target group was involved in all three.	Mental health and wellbeing	mHealth/ App	User-informed approaches should be used more often in development of digital health for refugees. Usability evaluation is ongoing. mHealth for mental health feedback from target group valuable and helped adapt design and content.
Dorfman et al. [62]	Refine and test a mobile-health behavioral cancer-pain-coping skills training protocol for women with breast cancer and pain from medically underserved areas.	Women with breast cancer and pain in medically underserved areas	Mixed Methods	3 focus groups (Phase 1) were used to refine the initial protocol. A single-arm pilot trial (Phase 2) was conducted to assess feasibility, acceptability and changes in outcomes.	Cancer	Telehealth	Appropriately adapted mobile-health technologies may provide an avenue to reach underserved patients and implement behavioral interventions to improve pain management.
Handley et al. [63]	Tailor an IT-enabled health communication program to promote a Diabetes Prevention Program (DPP)-induced concordant behavior change among the target group.	Postpartum Latina women with recent gestational diabetes	Qualitative	4 focus groups (n = 22 participants) and input from a regional consortium of health care providers, diabetes experts and health literacy practitioners informed the intervention development.	Pregnancy and/or postpartum	Telehealth	Systematic use of behavioral theory to inform intervention development could represent a strategy to develop health IT intervention tools to meet the needs of diverse populations.
Lee et al. [64]	Develop mobile text messaging intervention program, mScreening.	Young Korean American immigrant women	Qualitative	Guided by the Fogg behavior model, the mScreening intervention was developed through a series of focus groups.	HIV and/or other STDs	Text messages	This study demonstrated the processes of gathering culturally relevant information to develop a mobile phone text messaging intervention and incorporating the target population’s perspectives into the development of the intervention.
Liss et al. [65]	Design, implement and evaluate the ER Alert app, with patients from an underserved population.	Patients from a traditionally underserved population	Mixed Methods	Quantitative outcomes on app performance and qualitative data on patient user experience.	Overall lifestyle/health	mHealth/ App	The app had moderate sensitivity and high positive predictive value for identifying regional hospital visits.
Muroff et al. [66]	Translate and adapt an Addiction Comprehensive Health Enhancement Support system app for Spanish-speaking Latino adults with drug or alcohol disorders.	Spanish-speaking Latino/a adults	Quantitative	User data were collected for each unique user on a secure server, which captured login time stamps and services used within the CASA-CHESS app.	Mental health and wellbeing	mHealth/ App	Findings demonstrate the importance of social support in the four months following discharge for individuals with alcohol and other drug problems/mental disorders. Such evidence-based, theory-driven digital interventions can expand access to culturally and linguistically competent services.
Quarells et al. [67]	Adapt the Project UPLIFT providing mental health self-mangement for Black and Hispanic people with epilepsy (PWE).	Black and Hispanic people with epilepsy (PWE)	Qualitative	6 focus groups were conducted (4 PWE: People with Epilepsy; 2 MSP: Main Support Person). For PWE, there was one in-person focus group and three focus groups were conducted over the telephone.	Neurological conditions	Telehealth	The adaptation of Project UPLIFT for black and Hispanic people with epilepsy described here suggests that interventions can be successfully adapted to new populations or cultural settings through a careful and systematic adaptation process.
Sungur et al. [68]	Develop an oncological module aimed at increasing patient participation between older migrant patients with cancer and their health care professionals.	Older Turkish–Dutch and Moroccan–Dutch patients with cancer	Qualitative	In 5 cycles that engage key stakeholders in intervention development. Listen phase, Plan phase, Do phase, Study phase, Act phase.	Cancer	Website and informational videos	Websites and informational videos to inform older immigrant patients with cancer about health-related topics in their native language is a viable approach to increase the effectiveness of health communication for this target group.
Tanner et al. [69]	Describe the preliminary impact of weCare, an ongoing intervention to support HIV care linkage and retention for the target group.	Young racial/ethnic minority men who have sex with men (MSM) and transgender women with HIV (16–34 years)	Cohort study	Intervention staff and 91 intervention participants were interviewed on their experience with weCare.	HIV and/or other STDs	mHealth/ App and text messages	Social media is an important tool, especially for young MSM and transgender women, to support individual- and community- level health. It may also be a useful tool for improving engagement with biomedical HIV prevention tools.
**Type 4: Participation throughout development process (7 studies)**							
Brewer et al. [70]	Design and development of culturally relevant, cardiovascular health and wellness digital application.	African Americans in USA	Mixed Methods	Community-based participatory research approach.	Cardiac disease	mHealth/ App	Culturally relevant lifestyle interventions delivered by mobile health tools to comprehensively promote multiple cardiovascular risk factors through the Life’s Simple 7 framework can promote ideal CVH among African Americans, thereby advancing CVH equity.
Castillo et al. [71]	To adapt and translate an English-language pregnancy mobile health app to meet the language and cultural needs of Spanish-speaking Latino immigrants living in the United States.	Pregnancy and/or postpartum migrants and/or ethnic minorities	Qualitative	7-steps Transcreation Framework: (1) identify community infrastructure and engage partners; (2) specify theory; (3) identify multiple inputs for the new program; (4) design intervention prototype; (5) design study, methods and measures for community setting; (6) build community capacity for delivery; and (7) deliver the intervention.	Pregnancy and/or postpartum	mHealth/ App	Adaption of an mHealth pregnancy app to the needs of an emerging Latino community, by incorporating culturally sensitive Spanish language content while focusing on addressing existing health disparities.
DeCamp et al. [72]	Evaluate impact of Spanish-language test message intervention on infant emergency department use and well care and vaccine adherence.	Spanish-language (Latino) families in USA. 157 parent–child dyads.	RCT	Interactive text messages through the child’s first year of life, developed with the Latino Family Advisory Board. From grant proposal to implementation, meetings were conducted in Spanish and attended by 12 to15 members who were Latin American immigrant women.	Mental health and wellbeing	mHealth/ App and text messages	Reduced infant emergency department use and increased flu vaccine uptake, but no difference in welfare or overall immunization between intervention and control group.
Evans et al. [73]	Development of a theory-based mHealth intervention to promote HIV testing.	African community living in urban environments in the UK	Qualitative	Community-based social marketing process.	HIV and/or other STDs	mHealth/ App	Respondents highlighted a need for communities and professionals to work together to build a context of trust through co-location in, and co-involvement of, local communities which would, in turn, enhance confidence in, and support for, HIV testing activities of health professionals. Findings suggested that messages should: Avoid an exclusive focus on HIV, be tailored and personalized, come from a trusted source, allay fears and focus on support and health benefits.
Ospina-Pinillos et al. [74]	Cultural adaption and co-design of Mental Health E-clinic.	Spanish-speaking young people in Australia	Qualitative	Involvement of target group in phase 1 and 4. Involvement of Spanish speaking specialists and health professionals in 2 and 3.	Mental health and wellbeing	Website	Acceptability among target group is adequate. Further tailoring is needed.
Samkange-Zeeb et al. [75]	Development of an internet-based bilingual intelligent health assistant (IHA) and evaluate acceptance and usability.	Adults of Turkish origin living in Germany	Qualitative	Participation in all 4 phases of the development.	Overall lifestyle/health	Website and informational videos	Participants were ready to adopt and use IHA in their daily lives and thought that it needs to be tailored to their native language. However, they found some contents required cultural tailoring, while others did not.
Wang et al. [76]	Improve rate of mammography use.	Chinese women living in USA	Qualitative	Focus groups used to understand women’s views of breast cancer, attitudes toward mammography and preferred content. Meetings with community health leaders involved in cancer care and women’s health.	Cancer	Website and informational videos	Increase in screening intervention and knowledge, risk perception for breast cancer and benefit perception of mammography. Lower reported barriers after viewing.

## Data Availability

Keywords used for the systematic search process in this scoping review can be found under https://osf.io/dnp2j/ (accessed on 1 July 2023).
